# The epigenetic signature of chronic pain in the mouse brain

**DOI:** 10.1186/1744-8069-10-S1-O17

**Published:** 2014-12-15

**Authors:** Maral Tajerian, Sebastian Alvarado, Magali Millecamps, Moshe Szyf, Laura S Stone

**Affiliations:** 1 Department of Neurology and Neurosurgery, McGill University, Faculty of Medicine, 3801 University Street, Montreal, Quebec H3A 2B4, Canada; 2 Alan Edwards Centre for Research on Pain, McGill University, 740 Dr. Penfield Avenue, Montreal, Quebec H3A 0G1, Canada; 3 Department of Pharmacology and Therapeutics, McGill University, Faculty of Medicine, 3655 Promenade Sir William Osler, Montréal, Québec H3G 1Y6, Canada; 4 Sackler Program for Epigenetics & Developmental Psychobiology, McGill University, 3655 Promenade Sir William Osler, Montréal, Québec H3G 1Y6, Canada; 5 Faculty of Dentistry, McGill University, 3640 University Street, Montreal, Quebec H3A 0C7, Canada; 6 Department of Anesthesiology, Anesthesia Research Unit, McGill University, Faculty of Medicine, 3655 Promenade Sir William Osler, Montreal, Quebec H3G 1Y6, Canada

## Background

Peripheral nerve injury can be accompanied by long-term pain-related manifestations, such as affective and cognitive disturbances, suggesting the involvement of supraspinal mechanisms. One particular region of interest is the prefrontal cortex (PFC), an area implicated in depression, anxiety and cognitive impairment, all of which are frequently associated with chronic pain [1-4]. Clinically, pathological pain-related changes in the PFC in individuals with chronic low back pain can be reversed following effective pain management [5]. However, the mechanisms behind pain-induced brain plasticity remain poorly understood.

Epigenetics is a term used to describe modifications to genomic DNA that alter gene expression. DNA methylation is an epigenetic mechanism that is involved in gene regulation mainly by silencing promoter activity. We propose that long-term alterations in DNA methylation could provide a molecular substrate for chronic pain-related changes in the CNS, forming a ‘‘memory trace’’ for pain in the brain.

## Materials and methods

Spared nerve injury or sham surgery was performed in male CD1 mice at three months of age. Six months after injury, mechanical hypersensitivity was confirmed, brains were collected and DNA and RNA were extracted. Global DNA methylation was measured by the luminometric methylation assay in various brain regions, including the PFC. Promoter methylation of individual genes was assessed by sodium bisulfite sequencing and functionally validated using an *in vitro* promoter assay. Finally, mRNA levels of the target genes were measured by RT-PCR.

## Results

Six months following peripheral nerve injury, abnormal sensory thresholds and increased anxiety were accompanied by significant genomic DNA hypomethylation [6] and transcriptional reprogramming [7]. This was linked to the hypomethylation of individual genes, including (synaptotagmin 2) *syt2*, a known regulator of synaptic function. Furthermore, transcription of syt2 was regulated by differential methylation of its promoter in vitro and syt2 mRNA was upregulated in the PFC of injured animals. Thus chronic pain-induced changes in the PFC are detected long after the original injury, at a long distance from the site of injury.

## Conclusions

We show that peripheral injury produces long-term changes in the PFC methylome and propose that DNA methylation mediates the changes in brain structure and cortical function that are associated with chronic pain.

## Disclosures

The authors declare no competing interests.
